# Impact of socioeconomic status on patient characteristics and postoperative outcomes in colorectal cancer surgery: A retrospective cohort study

**DOI:** 10.1007/s00384-026-05149-z

**Published:** 2026-05-25

**Authors:** Nina Schraps, Amani Nassar, Siwen Zhang, Petro Zgurskyi, Marius Kemper, Eleftherios D. Papazoglou, Gerrit Wolters-Eisfeld, Anastasios D. Giannou, Jakob R. Izbicki, Thilo Hackert, Nathaniel Melling, Baris Mercanoglu

**Affiliations:** 1 Department of General, Visceral and Thoracic Surgery, University Medical Center Hamburg-Eppendorf, 20246 Hamburg, Germany; 2 Section of Molecular Immunology and Gastroenterology, I. Department of Medicine, University Medical Center Hamburg-Eppendorf, 20246 Hamburg, Germany

**Keywords:** Colorectal cancer, Colorectal cancer surgery, Socioeconomic status, Purchasing power index

## Abstract

**Background:**

Colorectal cancer remains among the most common malignancies worldwide and the second leading cause of cancer-related death. Prior studies suggest that socioeconomic deprivation is associated with higher incidence and poorer outcomes in colorectal cancer patients.

**Methods:**

We conducted a retrospective study of 476 patients who underwent colorectal cancer resection at a high-volume center in Germany between 2016 and 2023. Area-level socioeconomic status (SES) was estimated using a region-specific purchasing power index (PPI) derived from patients’ residential postal codes. We retrospectively examined whether SES was associated with mode of presentation, perioperative course, postoperative complications, and oncologic outcomes. In addition, a prespecified exploratory subgroup analysis was performed by comparing patients in the highest 20% (top) and lowest 20% (bottom) of the cohort according to PPI distribution.

**Results:**

In the overall cohort, higher area-level SES was associated with emergency surgery. In the subgroup analysis, the top SES subgroup was also associated with older age and postoperative complications. We further observed significant associations between the bottom SES subgroup and both younger age at diagnosis and higher body mass index (BMI). No statistically significant association between SES and overall or disease-free survival was observed in our patient cohort.

**Conclusions:**

In this single-center study, area-level SES was associated with distinct patient and perioperative profiles, but not with long-term oncologic outcomes. These findings suggest potentially actionable differences in risk profiles across SES strata, particularly regarding age at diagnosis, obesity, and complication burden.

**Supplementary Information:**

The online version contains supplementary material available at 10.1007/s00384-026-05149-z.

## Introduction

With about 1.8 million new cases and 900,000 deaths worldwide each year, colorectal cancer (CRC) is not only one of the most common tumor entities, but also the second most common cause of cancer-related deaths [1]. Recent epidemiological studies report a decline in CRC incidence in people over the age of 50 [2]. For reasons not yet fully understood, incidence among individuals younger than 50 years is increasing (early-onset colorectal cancer, EO-CRC), making it the leading cause of cancer-related deaths in men under 50 years of age [3–7]. Crucial for treatment and patient survival is the diagnosis at an early stage. CRC screening in Germany includes fecal occult blood tests and colonoscopies, which are offered from the age of 50 [8]. The treatment of colorectal cancer often requires a multidisciplinary approach that includes surgical resection, chemotherapy and radiation therapy [9]. Furthermore, advances in systemic therapy, including targeted therapy and immunotherapy, have substantially improved the treatment strategies for metastatic colorectal cancer [9].

The risk factors for the development of colorectal carcinomas can be categorized into two large groups of modifiable and non-modifiable risk factors. Non-modifiable risk factors for colorectal carcinomas include male gender, age, inflammatory bowel diseases, and hereditary mutations [10]. Examples of modifiable risk factors are patient diet, obesity [11, 12], smoking [13–15], diabetes [16–18] and alcohol consumption [19, 20]. Some of these risk factors are related to socioeconomic status (SES). For example, a higher SES is linked to higher alcohol consumption [21], while low SES is associated with smoking [22], and a higher prevalence of obesity [23] and diabetes [24]. It has been shown that the incidence and mortality rate of colorectal carcinomas are higher in socio-economically disadvantaged people [25, 26].

Our aim was to investigate possible associations between socioeconomic status and patient outcome after colorectal surgery for CRC.

## Materials and methods

### Study design and patient selection

**Fig. 1:**
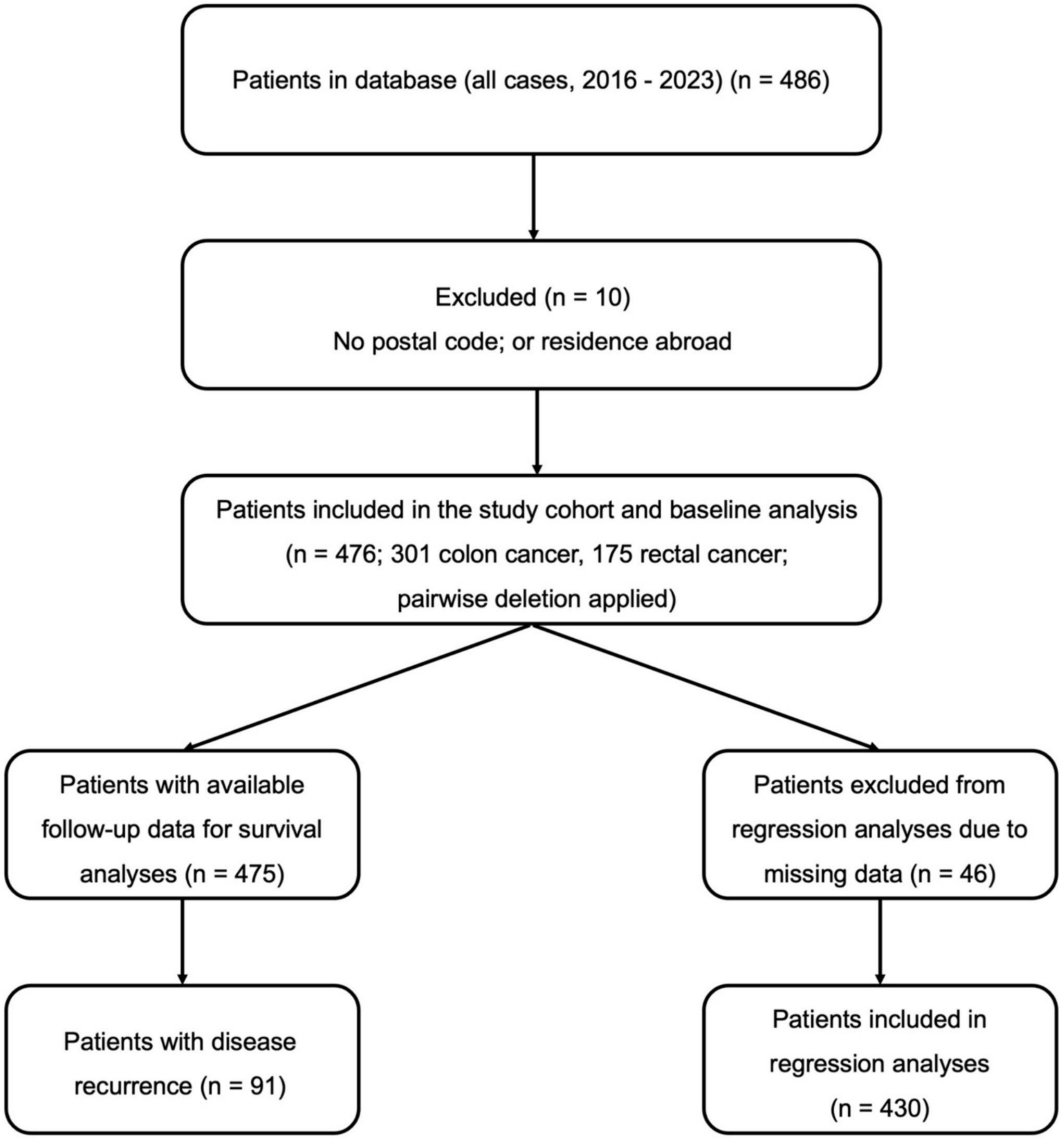
Flow diagram of patient selection

A retrospective analysis of a prospectively maintained database was conducted, including patients who underwent colorectal surgery for a first-time diagnosis of colorectal adenocarcinoma (UICC stage 0–IV) between January 2016 and December 2023 at the Department of General, Visceral and Thoracic Surgery, University Medical Center Hamburg-Eppendorf (UKE), Germany. Patients were eligible for inclusion if they had histologically confirmed primary colorectal adenocarcinoma, underwent surgical resection at our institution during the study period, and had available residential postal code information required for the assessment of area-level socioeconomic status. Patients were excluded if postal code information was missing, if their place of residence was outside Germany, or if they did not meet the predefined criteria of a first-time diagnosis of colorectal adenocarcinoma. A total of 486 patients were screened for eligibility, of whom 476 fulfilled the inclusion criteria and were included in the final study cohort, as illustrated in Fig. 1. All surgical procedures were performed by board-certified colorectal surgeons at a German Cancer Society (DKG)-certified colorectal cancer center. Emergency procedures were included and defined as operations performed within 6 h after the indication for surgery had been established. No age restrictions were applied. Both elective and emergency procedures were considered, including open, laparoscopic, and robot-assisted approaches. Neoadjuvant treatment was not used as an inclusion or exclusion criterion but was recorded as a clinical variable. The clinical database was approved by the Ethics Committee of the City of Hamburg, and written informed consent was obtained from all patients prior to inclusion in the clinical database. The study was conducted in accordance with the Declaration of Helsinki. The reporting of this study was performed in accordance with the Strengthening the Reporting of Observational Studies in Epidemiology (STROBE) guidelines [27]. The primary outcome was overall survival. Secondary outcomes included disease-free survival and postoperative complications. In addition, patient, operative, and tumor characteristics were analyzed.

### Data collection

Clinical data were collected and managed using REDCap (Research Electronic Data Capture) electronic data capture tools hosted at University Medical Center Hamburg-Eppendorf, Germany [28, 29]. REDCap is a secure, web-based software platform designed to support data capture for research studies. The preoperative Charlson Comorbidity Index (CCI) was assessed prior to surgery [30]. Postoperative complications were categorized using the Clavien-Dindo Classification System [31]. Preoperative diagnostic work-up was performed in accordance with the German S3 guideline for colorectal carcinoma [8]. Overall survival was defined as the time from the date of surgery to death from any cause, as recorded in the German National Population Register, or to the date of last contact with the population register (last follow-up). Disease-free survival was defined as the time from the date of surgery to the first documented disease recurrence, whether local or distant. The date of recurrence was defined as the earliest date of radiological or pathological evidence of relapse. Patients without an event were censored at the date of the last documented disease assessment or last clinical follow-up. Recurrence status was determined through systematic chart review, including external medical reports transmitted to our center. Only radiologically and/or pathologically confirmed recurrences were considered. A comprehensive overview of the variables and parameters recorded in our REDCap database is provided in Supplementary Table 1.

### Definition of socioeconomic status

Socioeconomic status (SES) was indicated using net income per capita per year, which has previously been validated as an appropriate proxy indicator of SES [32–36]. To determine individual income, we applied the regional Purchasing Power Index (PPI) as a surrogate parameter. Purchasing power reflects net income and was assigned by matching each patient’s residential postal code with the corresponding regional PPI, as described previously [32, 37–39]. PPI data were purchased from Michael Bauer Research GmbH and are based on data provided by the German Federal Statistical Office. For analysis, we used an annual per capita net income of €28,000 as the cutoff value, corresponding to the average annual net income per capita in Germany during the study period, based on data provided by the German Federal Statistical Office, thereby enabling a comparison between patients with below-average and above-average income levels. In addition, a prespecified exploratory subgroup analysis was performed by comparing patients in the highest 20% (top) and lowest 20% (bottom) quintiles of the cohort according to PPI distribution, in order to assess associations at the extremes of the socioeconomic spectrum as a complementary analysis. Quintiles are commonly used in income statistics to describe the distribution of income within populations and have also been applied in previous socioeconomic studies to compare the highest and lowest 20% of a cohort [40–44].

### Statistical analysis

Statistical analyses were performed in R version 4.3.0 (R Core Team, 2023) with the survival package. Assessment of the distribution of continuous variables was based on a combination of the Shapiro–Wilk test and graphical methods, including histograms, Q–Q plots, and boxplots. As continuous variables showed non-normal distributions, continuous and ordinal variables were summarized as median and interquartile range (IQR), and categorical variables were presented as absolute and relative frequencies. The chi-square test or Fisher’s exact test, as appropriate, was used to assess univariate associations between categorical variables, and the Mann–Whitney U test was applied for comparisons of continuous variables. Missing data in the baseline characteristics were handled using pairwise deletion. Overall survival (OS) and disease-free survival (DFS) were estimated using the Kaplan–Meier method, and survival differences between groups were compared with the log-rank test. Multivariable Cox proportional hazards regression models were employed to identify independent prognostic factors for overall survival and to account for potential confounding by clinically relevant baseline variables. Clinically relevant baseline variables with established or plausible prognostic relevance for overall survival after colorectal cancer surgery were predefined as candidate variables. Univariable Cox regression analyses were then performed as a variable-reduction step. Variables with *p* < 0.05 in the univariable analysis were subsequently entered into the multivariable Cox proportional hazards model. This approach was used to construct a parsimonious model and to limit the number of covariates in relation to the number of observed overall survival events, thereby reducing the risk of overfitting. In addition, multicollinearity among variables was assessed using variance inflation factors (VIFs), and no relevant collinearity was detected. For the regression analyses, a complete-case approach was applied, resulting in the inclusion of 430 of 476 patients (90.3%). Overall significance of variables was assessed using likelihood ratio tests, and hazard ratios (HR) with 95% confidence intervals (CI) were reported. A two-sided *p*-value < 0.05 was considered statistically significant.

## Results

### Patient flow and study cohort

A total of 486 patients were recorded in the database. 10 patients were excluded because of missing postal code information or residence outside Germany. The final study cohort therefore comprised 476 patients, including 301 patients with colon cancer and 175 patients with rectal cancer. These 476 patients were included in the baseline analysis presented in Table 1. Follow-up data were available for 475 patients, who were therefore included in the survival analyses. Overall, 91 patients experienced disease recurrence. The proportion of missing baseline data was low, with at most 35 of 476 observations missing for any given variable (7.4%). Supplementary Tables 2 and 3 provide summaries of missing data. For the regression analyses, a complete-case approach was applied; 46 patients were excluded because of missing data, resulting in a complete-case cohort of 430 patients included in the regression models. Figure 1 shows the flowchart of patient selection.

**Table 1:** Associations between socioeconomic status and overall cohort characteristics

Characteristics	All patients (*n* = 476)	Low SES (*n* = 202)	High SES (*n* = 274)	*p*-value
Purchasing Power (EUR [IQR])	28,721.00 [26514.75, 32207.00]	26,061.00 [24400.00, 27308.50]	31,605.00 [29531.00, 34066.00]	n.a
Age (years [IQR])	66.00 [55.00, 75.00]	64.00 [56.00, 74.00]	66.00 [55.00, 76.75]	0.232
Gender [n (%)]				
Female	198 (41.6)	84 (41.6)	114 (41.6)	1
Male	278 (58.4)	118 (58.4)	160 (58.4)	
BMI (kg/m^2^ [IQR])	24.69 [21.80, 27.89]	24.99 [21.84, 28.08]	24.58 [21.80, 27.45]	0.373
Charlson Comorbidity Index (IQR)	3.00 [1.00, 4.00]	3.00 [1.25, 4.00]	3.00 [1.00, 4.00]	0.931
ECOG [n (%)]				
0	354 (77.3)	155 (80.3)	199 (75.1)	**0.042**
1	66 (14.4)	27 (14.0)	39 (14.7)	
2	21 (4.6)	10 (5.2)	11 (4.2)	
3	15 (3.3)	1 (0.5)	14 (5.3)	
4	2 (0.4)	0 (0.0)	2 (0.8)	
ASA [n (%)]				
I	14 (3.0)	7 (3.5)	7 (2.6)	0.510
II	189 (40.5)	82 (41.0)	107 (40.1)	
III	222 (47.5)	98 (49.0)	124 (46.4)	
IV	41 (8.8)	13 (6.5)	28 (10.5)	
V	1 (0.2)	0 (0.0)	1 (0.4)	
Diabetes [n (%)]				
No	399 (83.8)	174 (86.1)	225 (82.1)	0.293
Yes	77 (16.2)	28 (13.9)	49 (17.9)	
Nicotine abuse [n (%)]				
No	334 (70.2)	143 (70.8)	191 (69.7)	0.877
Yes	142 (29.8)	59 (29.2)	83 (30.3)	
Urgency of surgery [n (%)]				
Elective	407 (85.5)	183 (90.6)	224 (81.8)	**0.010**
Emergency	69 (14.5)	19 (9.4)	50 (18.2)	
Surgical approach [n (%)]				
Open	212 (44.5)	82 (40.6)	130 (47.4)	0.254
Laparoscopic	119 (25.0)	57 (28.2)	62 (22.6)	
Robotic	145 (30.5)	63 (31.2)	82 (29.9)	
Conversion [n (%)]				
No	438 (92.0)	187 (92.6)	251 (91.6)	0.830
Yes	38 (8.0)	15 (7.4)	23 (8.4)	
Tumor Location [n (%)]				
Colon	301 (63.2)	126 (62.4)	175 (63.9)	0.812
Rectum	175 (36.8)	76 (37.6)	99 (36.1)	
Procedure [n (%)]				
Anterior rectum resection	129 (27.2)	57 (28.4)	72 (26.3)	0.492
Left hemicolectomy	123 (25.9)	46 (22.9)	77 (28.1)	
Proctocolectomy	20 (4.2)	6 (3.0)	14 (5.1)	
Rectum extirpation	39 (8.2)	18 (9.0)	21 (7.7)	
Right hemicolectomy	164 (34.5)	74 (36.8)	90 (32.8)	
Operative Time (minutes [IQR])	232.00 [177.50, 286.00]	235.00 [175.50, 307.25]	229.00 [182.00, 274.00]	0.443
UICC [n (%)]				
0	8 (1.7)	3 (1.5)	5 (1.8)	0.086
I	101 (21.2)	52 (25.7)	49 (17.9)	
II	132 (27.7)	44 (21.8)	88 (32.1)	
III	147 (30.9)	65 (32.2)	82 (29.9)	
IV	88 (18.5)	38 (18.8)	50 (18.2)	
pT [n (%)]				
T0	11 (2.3)	5 (2.5)	6 (2.2)	0.288
T1	42 (8.8)	16 (7.9)	26 (9.5)	
T2	96 (20.2)	50 (24.8)	46 (16.8)	
T3	235 (49.4)	96 (47.5)	139 (50.7)	
T4	92 (19.3)	35 (17.3)	57 (20.8)	
pN [n (%)]				
N0	261 (54.8)	106 (52.5)	155 (56.6)	0.714
N1	143 (30.0)	62 (30.7)	81 (29.6)	
N2a	40 (8.4)	20 (9.9)	20 (7.3)	
N2b	32 (6.7)	14 (6.9)	18 (6.6)	
pM [n (%)]				
M0	351 (73.9)	146 (72.3)	205 (75.1)	0.159
M1a	71 (14.9)	37 (18.3)	34 (12.5)	
M1b	22 (4.6)	10 (5.0)	12 (4.4)	
M1c	31 (6.5)	9 (4.5)	22 (8.1)	
Resection Status [n (%)]				
R0	444 (95.7)	190 (95.0)	254 (96.2)	0.477
R1	19 (4.1)	9 (4.5)	10 (3.8)	
R2	1 (0.2)	1 (0.5)	0 (0.0)	
Neoadjuvant Chemotherapy [n (%)]				
No	395 (83.0)	160 (79.2)	235 (85.8)	0.079
Yes	81 (17.0)	42 (20.8)	39 (14.2)	
Neoadjuvant Radiotherapy [n (%)]				
No	420 (88.2)	172 (85.1)	248 (90.5)	0.099
Yes	56 (11.8)	30 (14.9)	26 (9.5)	
Adjuvant Chemotherapy [n (%)]				
No	237 (49.8)	95 (47.0)	142 (51.8)	0.346
Yes	239 (50.2)	107 (53.0)	132 (48.2)	
Adjuvant Radiotherapy [n (%)]				
No	451 (94.7)	194 (96.0)	257 (93.8)	0.381
Yes	25 (5.3)	8 (4.0)	17 (6.2)	
Clavien-Dindo Classification [n (%)]				
I	45 (18.4)	24 (22.6)	21 (15.2)	0.170
II	61 (25.0)	26 (24.5)	35 (25.4)	
III a	20 (8.2)	13 (12.3)	7 (5.1)	
III b	60 (24.6)	23 (21.7)	37 (26.8)	
IV a	26 (10.7)	10 (9.4)	16 (11.6)	
IV b	6 (2.5)	1 (0.9)	5 (3.6)	
V	26 (10.7)	9 (8.5)	17 (12.3)	
Length of hospital stay (days [IQR])	9.00 [7.00, 16.00]	9.00 [6.00, 16.00]	10.00 [7.00, 16.00]	0.441

Data are presented as median (interquartile range [IQR]) or n (%). Bold type indicates *p*-values < 0.05, facilitating identification of statistically significant results

Abbreviations: n.a., not applicable, as purchasing power was the grouping variable; *ASA*, American Society of Anesthesiologists; *BMI*, body mass index; *ECOG*, Eastern Cooperative Oncology Group; *UICC*, Union for International Cancer Control

### Associations of socioeconomic status and characteristics of the patient cohort

Table 1 shows the relationship between SES and clinical, as well as histopathological characteristics of the patient cohort. The median purchasing power of the 476 patients included in this study was EUR 28,721 (IQR 26514.75—32,207.00). A total of 202 (42.4%) patients were classified as low SES, defined as an estimated net annual income per capita below EUR 28,000. The high SES group included 274 patients (57.6%) with an estimated net annual income per capita of more than EUR 28,000. The median age of all patients was 66 years (IQR 55—75). No significant difference in age was observed between the low and high SES groups (*p* = 0.232).

The primary tumor was located in the colon in 301 (63.2%) patients. A total of 175 (36.8%) patients had rectal cancer. There was no association between socioeconomic status and the localization of the primary tumor (*p* = 0.812).

No significant differences between the low and high SES group were found regarding the Charlson Comorbidity Index (*p* = 0.931) as a tool for measuring comorbidity and predicting long-term mortality. The perioperative risk measured according to the ASA (American Society of Anesthesiologists) classification also showed no significant link with SES (*p* = 0.510). However, low SES was associated with a significantly better ECOG Performance Status compared with high SES (*p* = 0.042).

Patients with a high SES were significantly more likely to have emergency operations (*p* = 0.010). Open surgery was performed in 212 patients (44.5%), laparoscopic surgery in 119 patients (25.0%) and robotic surgery in 145 patients (30.5%). Conversion to open surgery was seen in 38 patients (8.0%). No significant differences were found between the low and high SES groups in terms of surgical approach (*p* = 0.254) or conversion rate (*p* = 0.830). Furthermore, there were no differences in the procedure performed (*p* = 0.492) or the time of the operation (*p* = 0.443). Minor complications (Clavien-Dindo Grade I–IIIa) occurred in 126 patients (26.47%). Major postoperative complications (Clavien-Dindo Grade IIIb–IV) were seen in 92 patients (19.33%). Lastly, 26 patients (5.46%) died during the hospital stay (Clavien-Dindo Grade V). There were no significant differences in severity and frequency of postoperative complications (Clavien-Dindo Grade) between high and low SES (*p* = 0.170). The length of the hospital stay did not differ in regard to the socioeconomic status (*p* = 0.441).

In the overall cohort, multivariable logistic regression analyses were performed with emergency surgery and postoperative complications as dependent variables and SES, age, and Charlson Comorbidity Index as independent variables. After adjustment, higher SES remained independently associated with emergency surgery (high SES: OR = 2.15, 95% CI: 1.24—3.87, *p* = 0.01; Suppl. Table 4). In contrast, higher SES was not independently associated with postoperative complications after adjustment for age and Charlson Comorbidity Index (high SES: OR = 1.31, 95% CI: 0.78—2.19, *p* = 0.31; Suppl. Table 5).

The UICC tumor stage did not significantly vary between low and high SES groups (*p* = 0.086). Furthermore, there was no difference in histopathological parameters including pathological tumor stage (*p* = 0.288), lymph node stage (*p* = 0.714), distant metastasis (*p* = 0.159) and resection status (*p* = 0.477).

Additionally, no discrepancies were found in the frequency of neoadjuvant (*p* = 0.079) or adjuvant (*p* = 0.346) chemotherapy, as well as neoadjuvant (*p* = 0.099) or adjuvant (*p* = 0.381) radiotherapy between high and low SES.

There were no significant associations between socioeconomic status and gender (*p* = 1), nicotine abuse (*p* = 0.877), BMI (*p* = 0.373), or diabetes (*p* = 0.293).

### Socioeconomic status and patient survival

**Fig. 2:**
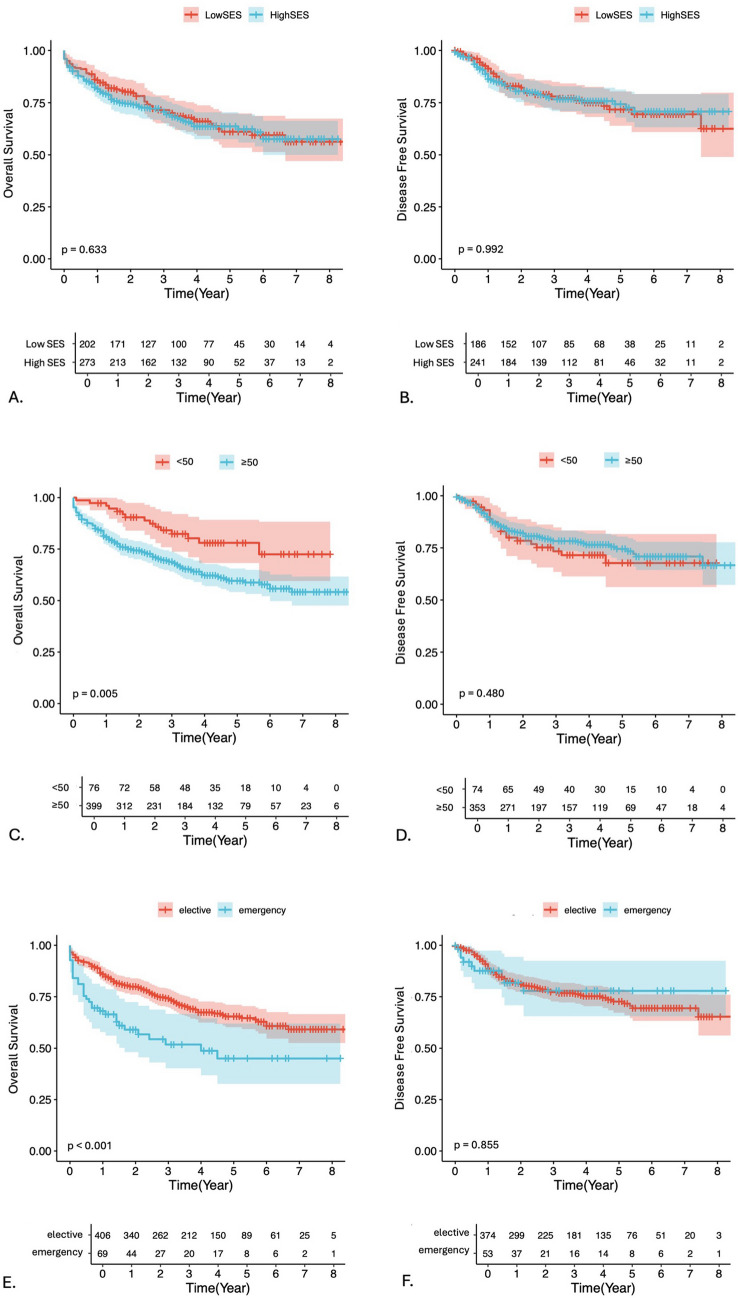
The Kaplan–Meier survival curves show the association between overall survival as well as disease-free survival and SES (**A-B**), age above or below 50 years (**C-D**) and urgency (**E–F**) in colorectal cancer patients

The findings of the univariate and multivariate analysis are shown in Table 2. The analysis revealed no significant association between socioeconomic status and overall survival (OS) (*p* = 0.633; Fig. 2A), as well as disease-free survival (DFS) (*p* = 0.992; Fig. 2B). There was a significant inverse correlation between age, stratified in 10-year intervals, and patient outcome in both univariate (*p* < 0.001) and multivariate analysis (*p* = 0.001). Univariate analysis further revealed that patients with early-onset colorectal cancer, which generally refers to individuals diagnosed with colorectal cancer before the age of 50, had significantly better overall survival than patients over the age of 50 (*p* = 0.005; Fig. 1C). This was also confirmed in the multivariate analysis (*p* = 0.002). There was no association between the age above or below 50 and disease-free survival (*p* = 0.480; Fig. 2D). Emergency presentations were associated with poorer OS (*p* < 0.001; Fig. 2E), but not with DFS (*p* = 0.855; Fig. 2F). In addition, univariate analysis showed a significant association between UICC (*p* < 0.001; Suppl. Figure 1B), surgical approach (*p* < 0.001), the procedure performed (*p* = 0.02), ECOG status (*p* < 0.001), ASA physical status (*p* < 0.001), and diabetes (*p* = 0.027).

**Table 2:** Univariable and Multivariable Cox Regression Analyses of Overall Survival

Characteristics	All patients (*n* = 430)	Univariate Analysis		Multivariate Analysis	
		Restricted Mean Survival Time in months (SE)	*p*-value	Hazard Ratio (95% CI)	*p*-value
Socioeconomic Status (%)					
Low SES	185 (43.0)	73 (3.41)	0.953		0.821
High SES	245 (57.0)	73 (3.03)		0.96 (0.66—1.39)	
Age (%)					
< 50	69 (16.0)	87 (4.68)	**0.005**	1.79 (0.9—3.59)	**0.002**
≥ 50	361 (84.0)	70 (2.52)			
Gender (%)					
Female	181 (42.1)	75 (3.42)	0.406		
Male	249 (57.9)	71 (3)			
BMI (%)					
< 25	228 (53.0)	72 (3.02)	0.732		
≥ 25	202 (47.0)	74 (3.15)			
Charlson Comorbidity Index (%)					
< 3	190 (44.2)	82 (2.96)	** < 0.001**		**0.005**
≥ 3	240 (55.8)	66 (3.21)		1.65 (1.06—2.58)	
ECOG (%)					
0	335 (77.9)	78 (2.43)	** < 0.001**		** < 0.001**
1	61 (14.2)	65 (5.93)		1.04 (0.63––1.70)	
2	20 (4.7)	42 (11.37)		4.41 (2.24—8.67)	
3	12 (2.8)	51 (15.36)		1.83 (0.75—4.49)	
4	2 (0.5)	8 (1.77)		1.97 (0.39—9.97)	
ASA (%)					
I	13 (3.0)	80 (12.15)	** < 0.001**		**0.001**
II	178 (41.4)	85 (3.09)		0.88 (0.26—3.00)	
III	204 (47.4)	68 (3.31)		1.57 (0.47—5.26)	
IV	34 (7.9)	44 (8.63)		3.33 (0.93—11.97)	
V	1 (0.2)	0 (0)		21.61 (1.87—249.45)	
Diabetes (%)					
No	362 (84.2)	75 (2.41)	**0.027**		0.077
Yes	68 (15.8)	61 (6.15)		1.34 (0.83—2.17)	
Nicotine abuse (%)					
No	296 (68.8)	74 (2.67)	0.446		
Yes	134 (31.2)	70 (4.25)			
Urgency of surgery (%)					
Elective	379 (88.1)	75 (2.35)	**0.004**		0.189
Emergency	51 (11.9)	58 (7.31)		1.37 (0.79—2.38)	
Surgical approach (%)					
Open	182 (42.3)	61 (3.72)	** < 0.001**		** < 0.001**
Laparoscopic	110 (25.6)	77 (4.06)		0.96 (0.60—1.52)	
Robotic	138 (32.1)	86 (3.47)		0.65 (0.38—1.11)	
Conversion (%)					
No	395 (91.9)	72 (2.37)	0.306		
Yes	35 (8.1)	81 (7.49)			
Tumor Location (%)					
Colon	270 (62.8)	74 (2.8)	0.486		
Rectum	160 (37.2)	72 (3.72)			
Procedure (%)					
Anterior rectum resection	118 (27.4)	78 (4.11)	**0.02**		0.083
Left hemicolectomy	115 (26.7)	75 (4.17)		0.68 (0.39—1.19)	
Proctocolectomy	18 (4.2)	67 (11.26)		1.41 (0.58—3.41)	
Rectum extirpation	35 (8.1)	52 (8.31)		1.51 (0.77—2.97)	
Right hemicolectomy	144 (33.5)	73 (3.96)		0.78 (0.46—1.33)	
UICC (%)					
Early	219 (50.9)	85 (2.78)	** < 0.001**		** < 0.001**
Mid	136 (31.6)	70 (4.05)		2.29 (1.48—3.54)	
Late	75 (17.4)	42 (5.65)		6.23 (3.77—10.31)	
Resection Status (%)					
R0	413 (96.0)	75 (2.28)	** < 0.001**		**0.01**
R1	16 (3.7)	27 (8.32)		2.48 (1.19—5.17)	
R2	1 (0.2)	105 (0)		-	
Neoadjuvant Chemotherapy (%)					
No	355 (82.6)	73 (2.47)	0.929		
Yes	75 (17.4)	73 (5.6)			
Neoadjuvant Radiotherapy (%)					
No	377 (87.7)	72 (2.41)	0.557		
Yes	53 (12.3)	78 (6.12)			

Data are presented as *n* (%). Bold type indicates *p*-values < 0.05, facilitating identification of statistically significant results. The multivariable analysis included only variables significant in the univariable analysis. For the regression analyses, complete-case analysis was performed, resulting in the inclusion of 430 of 476 patients (90.3%)

Abbreviations: *ASA*, American Society of Anesthesiologists; *BMI*, body mass index; *CI*, confidence interval; *ECOG*, Eastern Cooperative Oncology Group; *SE*, standard error; *UICC*, Union for International Cancer Control

In the multivariate analysis, overall survival was independently associated with age (*p* = 0.002), surgical approach (*p* < 0.001), UICC (*p* < 0.001), resection status (*p* = 0.01), ECOG status (*p* < 0.001), ASA physical status (*p* = 0.001) and Charlson Comorbidity Index (*p* = 0.005). Neoadjuvant radio- and/or chemotherapy, tumor location, conversion to open surgery, gender, nicotine abuse (*p* > 0.05 each), or BMI (*p* = 0.717; Suppl. Figure 1A) were not significantly associated with overall survival.

### Associations between the top and bottom SES and patient characteristics

Due to the relatively homogeneous income structure in the study population (median purchasing power of EUR 28,721; IQR 26514.75—32,207.00), the patient cohort was further subdivided according to the purchasing power of the top 20% compared with the bottom 20%, and a corresponding subgroup analysis was performed (95 patients each) (Table 3). Significant differences were seen in the age, BMI, Charlson Comorbidity Index, Clavien-Dindo Classification, and urgency. The median age was 64 years (IQR 55.0—71.5) in the bottom group and 73 (IQR 56.5—81.0) in the top group. The bottom group was significantly younger than the top group (*p* < 0.001). The bottom group further had a significant higher BMI of 25.28 kg/m^2^ (IQR 22.54—28.42) compared to the top group with 23.82 kg/m^2^ (IQR 22.22—26.63) (*p* = 0.033). A higher Charlson Comorbidity Index (*p* = 0.041) was seen in patients of the top group. Regarding the Clavien-Dindo Classification, minor complications were particularly prevalent in the bottom group, whereas severe complications of grade IIIb-V were more common in the top group (*p* = 0.041). Particularly striking was further the significantly higher proportion of emergency procedures in the top group (*p* < 0.001).

**Table 3:** Associations between top versus bottom socioeconomic status subgroups and cohort characteristics

Characteristics	All patients (*n* = 190)	Bottom SES (*n* = 95)	Top SES (*n* = 95)	*p-*value
Purchasing Power (EUR [IQR])	29,384.50 [24361.00, 35,519.00]	24,361.00 [23751.50, 25,352.50]	35,519.00 [34066.00, 39,683.00]	n.a
Age (years [IQR])	68.00 [55.25, 77.75]	64.00 [55.00, 71.50]	73.00 [56.50, 81.00]	** < 0.001**
Gender [n (%)]				
Female	82 (43.2)	43 (45.3)	39 (41.1)	0.66
Male	108 (56.8)	52 (54.7)	56 (58.9)	
BMI (kg/m^2^ [IQR])	24.57 [22.28, 27.70]	25.28 [22.54, 28.42]	23.82 [22.22, 26.63]	**0.033**
Charlson Comorbidity Index (IQR)	3.00 [2.00, 4.00]	3.00 [1.00, 4.00]	3.00 [2.00, 5.00]	**0.041**
ECOG [n (%)]				
0	140 (77.8)	73 (81.1)	67 (74.4)	0.078
1	23 (12.8)	12 (13.3)	11 (12.2)	
2	9 (5.0)	5 (5.6)	4 (4.4)	
3	7 (3.9)	0 (0.0)	7 (7.8)	
4	1 (0.6)	0 (0.0)	1 (1.1)	
ASA [n (%)]				
I	4 (2.1)	2 (2.1)	2 (2.1)	0.430
II	75 (39.7)	40 (42.1)	35 (37.2)	
III	89 (47.1)	46 (48.4)	43 (45.7)	
IV	21 (11.1)	7 (7.4)	14 (14.9)	
Diabetes [n (%)]				
No	163 (85.8)	82 (86.3)	81 (85.3)	1
Yes	27 (14.2)	13 (13.7)	14 (14.7)	
Nicotine abuse [n (%)]				
No	146 (76.8)	68 (71.6)	78 (82.1)	0.122
Yes	44 (23.2)	27 (28.4)	17 (17.9)	
Urgency of surgery [n (%)]				
Elective	156 (82.1)	88 (92.6)	68 (71.6)	** < 0.001**
Emergency	34 (17.9)	7 (7.4)	27 (28.4)	
Surgical approach [n (%)]				
Open	88 (46.3)	40 (42.1)	48 (50.5)	0.225
Laparoscopic	43 (22.6)	20 (21.1)	23 (24.2)	
Robotic	59 (31.1)	35 (36.8)	24 (25.3)	
Conversion [n (%)]				
No	174 (91.6)	86 (90.5)	88 (92.6)	0.794
Yes	16 (8.4)	9 (9.5)	7 (7.4)	
Tumor Location [n (%)]				
Colon	114 (60.0)	55 (57.9)	59 (62.1)	0.657
Rectum	76 (40.0)	40 (42.1)	36 (37.9)	
Procedure [n (%)]				
Anterior rectum resection	58 (30.7)	32 (34.0)	26 (27.4)	0.427
Left hemicolectomy	49 (25.9)	23 (24.5)	26 (27.4)	
Proctocolectomy	9 (4.8)	2 (2.1)	7 (7.4)	
Rectum extirpation	16 (8.5)	9 (9.6)	7 (7.4)	
Right hemicolectomy	57 (30.2)	28 (29.8)	29 (30.5)	
Operative Time (minutes [IQR])	239.00 [181.50, 302.00]	251.00 [181.50, 315.50]	235.00 [182.00, 272.50]	0.125
UICC [n (%)				
0	3 (1.6)	2 (2.1)	1 (1.1)	0.241
I	35 (18.4)	23 (24.2)	12 (12.6)	
II	53 (27.9)	22 (23.2)	31 (32.6)	
III	55 (28.9)	26 (27.4)	29 (30.5)	
IV	44 (23.2)	22 (23.2)	22 (23.2)	
pT [n (%)]				
T0	5 (2.6)	3 (3.2)	2 (2.1)	0.317
T1	14 (7.4)	8 (8.4)	6 (6.3)	
T2	34 (17.9)	22 (23.2)	12 (12.6)	
T3	98 (51.6)	45 (47.4)	53 (55.8)	
T4	39 (20.5)	17 (17.9)	22 (23.2)	
pN [n (%)]				
N0	101 (53.2)	50 (52.6)	51 (53.7)	0.402
N1	62 (32.6)	28 (29.5)	34 (35.8)	
N2a	11 (5.8)	6 (6.3)	5 (5.3)	
N2b	16 (8.4)	11 (11.6)	5 (5.3)	
pM [n (%)]				
M0	132 (69.5)	63 (66.3)	69 (72.6)	0.067
M1a	35 (18.4)	20 (21.1)	15 (15.8)	
M1b	8 (4.2)	7 (7.4)	1 (1.1)	
M1c	15 (7.9)	5 (5.3)	10 (10.5)	
Resection Status [n (%)]				
R0	175 (95.6)	87 (93.5)	88 (97.8)	0.300
R1	8 (4.4)	6 (6.5)	2 (2.2)	
Neoadjuvant chemotherapy [n (%)]				
No	158 (83.2)	75 (78.9)	83 (87.4)	0.175
Yes	32 (16.8)	20 (21.1)	12 (12.6)	
Neoadjuvant radiotherapy [n (%)]				
No	169 (88.9)	81 (85.3)	88 (92.6)	0.165
Yes	21 (11.1)	14 (14.7)	7 (7.4)	
Adjuvant chemotherapy [n (%)]				
No	90 (47.4)	43 (45.3)	47 (49.5)	0.663
Yes	100 (52.6)	52 (54.7)	48 (50.5)	
Adjuvant radiotherapy [n (%)]				
No	181 (95.3)	92 (96.8)	89 (93.7)	0.495
Yes	9 (4.7)	3 (3.2)	6 (6.3)	
Clavien-Dindo Classification [n (%)]				
I	19 (18.8)	13 (25.0)	6 (12.2)	**0.041**
II	21 (20.8)	9 (17.3)	12 (24.5)	
III a	9 (8.9)	8 (15.4)	1 (2.0)	
III b	26 (25.7)	13 (25.0)	13 (26.5)	
IV a	12 (11.9)	6 (11.5)	6 (12.2)	
IV b	3 (3.0)	1 (1.9)	2 (4.1)	
V	11 (10.9)	2 (3.8)	9 (18.4)	
Length of hospital stay (days [IQR])	10.00 [7.00, 15.50]	9.00 [6.00, 14.00]	10.50 [7.00, 16.00]	0.345

Data are presented as median (interquartile range [IQR]) or n (%). Bold type indicates *p*-values < 0.05, facilitating identification of statistically significant results

Abbreviations: n.a., not applicable, as purchasing power was the grouping variable; *ASA*, American Society of Anesthesiologists; *BMI*, body mass index; *ECOG*, Eastern Cooperative Oncology Group; *UICC*, Union for International Cancer Control

In the top and bottom SES subgroups, multivariable logistic regression analyses were performed for emergency surgery and postoperative complications, including SES, age, and Charlson Comorbidity Index as independent variables. After adjustment, higher SES remained independently associated with emergency surgery (top SES: OR = 5.05, 95% CI: 2.15—13.38, *p* < 0.001; Suppl. Table 6), whereas no independent association was observed between higher SES and postoperative complications (top SES: OR = 1.29, 95% CI: 0.56—3.01, *p* = 0.55; Suppl. Table 7).

**Fig. 3:**
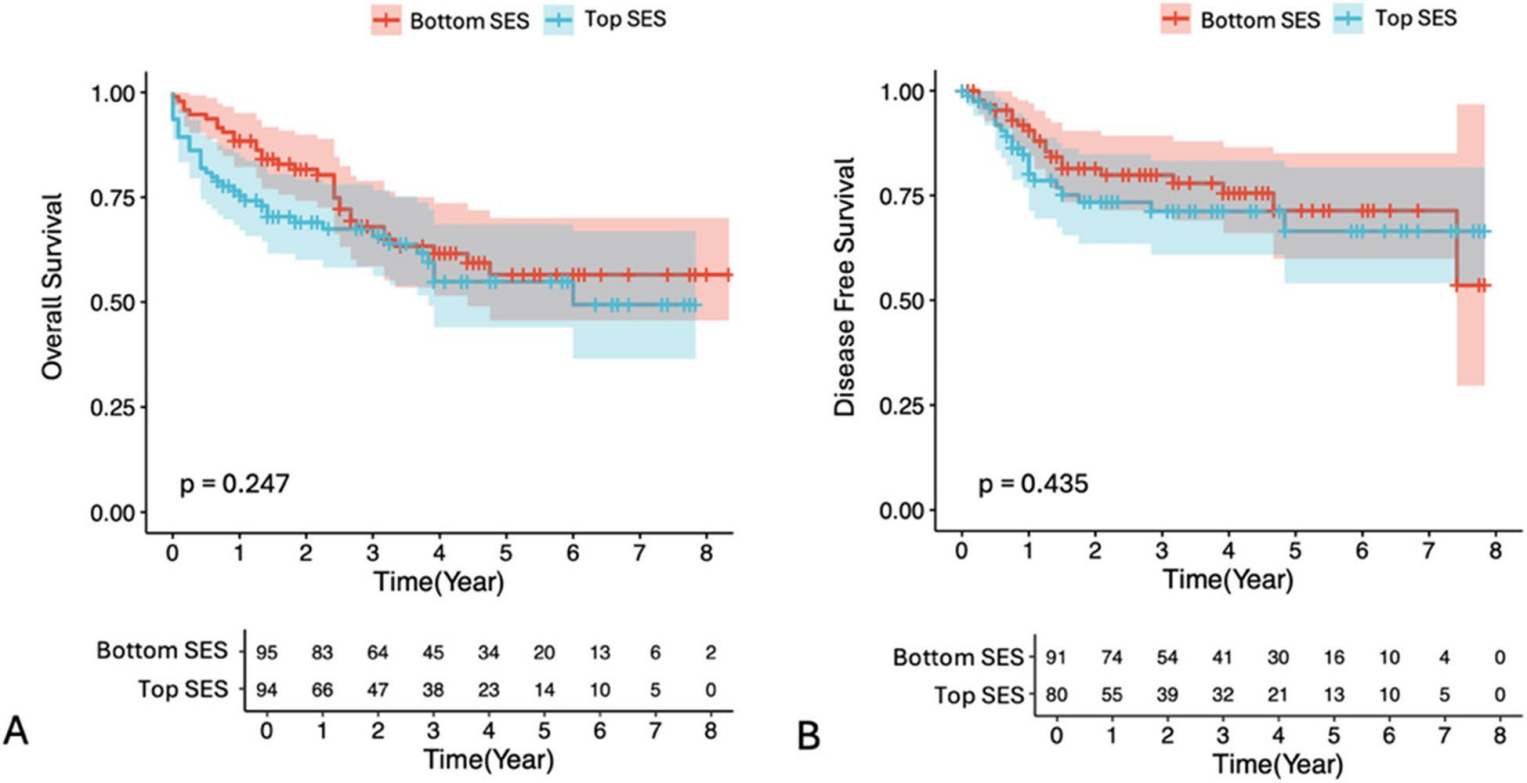
Associations between top and bottom SES and overall survival (**A**) and disease-free survival (**B**) in colorectal cancer patients

The subgroup analysis showed no significant association between socioeconomic status and overall survival (*p* = 0.247; Fig. 3A) or disease-free survival (*p* = 0.435; Fig. 3B).

## Discussion

In this retrospective, single-center study conducted at a high-volume center in Germany, we investigated associations between area-level SES and clinical as well as histopathological data from colorectal cancer patients undergoing surgery. In the overall cohort, higher area-level SES was associated with emergency surgery. In addition, in the prespecified exploratory subgroup analysis, the top SES subgroup was associated with older age and postoperative complications. We further observed significant associations between the bottom SES subgroup and both younger age at diagnosis and higher BMI. No statistically significant association between area-level SES and overall or disease-free survival was observed in our patient cohort.

A key finding of our study was the significant difference in age at diagnosis between the top and bottom 20% of the patient cohort. The bottom SES subgroup was significantly younger than the top SES subgroup. Furthermore, our analysis revealed that age was an independent prognostic factor for OS. Patients with EO-CRC had a better overall survival than patients over the age of 50. In accordance with our data, a study by Zhu et al., which analyzed data from 17 cancer registries in the USA with a total of 761,697 patient records on CRC, described a higher proportion of younger patients with low median household income [45]. The existing literature investigating the relationship between EO-CRC and patient survival is largely consistent with our findings. In line with our findings, Wang et al. identified age over 50 as an independent predictor of poor prognosis, whereas patients with EO-CRC demonstrated better survival despite presenting at a later stage and with more aggressive pathological features [46]. This was also confirmed by Abdelsattar et al., who reported better 5-year disease-specific survival in EO-CRC patients regardless of more advanced tumor stage at diagnosis [47]. Kolarich et al. showed better short- and long-term outcomes for patients with early-onset rectal cancer [48]. Possible explanations include fewer comorbidities, differences in treatment strategies with more aggressive therapy, and distinct tumor mutation profiles [49]. In a patient cohort of 45,660 EO-CRC patients, Ko et al. showed that patients with the lowest neighborhood SES were more likely to present with metastasis and had lower survival compared to the group in the highest neighborhood SES [50]. This was also confirmed by Salem et al. in a patient cohort of 30,903 demonstrating a worse outcome in patients with EO-CRC with a low SES [51]. While an overall increase in the incidence of EO-CRC has been observed, the reasons for this increase remain largely unclear [7, 52, 53]. Previous studies show a link between lifestyle factors and the increased incidence of EO-CRC, including a higher BMI, dietary factors, or metabolic syndrome [52, 54]. Our data showed a significantly higher BMI in the bottom SES subgroup compared to the top SES subgroup, which may represent a potential risk factor associated with the occurrence of EO-CRC.

Another unexpected finding was that high area-level SES was associated with an increased rate of emergency surgery and poorer ECOG Performance Status. The top SES subgroup further showed a higher rate of major postoperative complications according to the Clavien-Dindo classification and a higher Charlson Comorbidity Index. In contrast, previous literature has tended to associate a low SES with more comorbidities and an increased risk of emergency presentation [55–58]. The higher rate of emergency surgeries may be due to residual confounding or to other factors not measured in our analysis. After adjustment for age and Charlson Comorbidity Index, higher area-level SES was not independently associated with postoperative complications in our study. The significantly higher age of the top SES subgroup compared with the bottom SES subgroup may therefore explain the higher rate of postoperative complications. That patients with emergency surgery had a worse OS than patients with elective surgery is consistent with the literature, which has shown higher 30-day and 1-year mortality in case of emergency presentation [55].

In our study, no statistically significant association was observed between area-level SES and either OS or DFS. This is in contrast to many international studies, including studies from Canada [41, 59], the Netherlands [33], the United Kingdom [60, 61], the USA [45, 62] and France [63], which show a link between low SES and poor OS as well as disease-specific survival. For example, van den Berg et al. demonstrated an independent association between lower SES and a poor cancer specific survival after analyzing 965 patients undergoing curative surgery in the Netherlands [33]. Zhu et al. showed that a higher Median Household Income improved OS regardless of the stage of CRC, including more than 700,000 patient records from 17 cancer registries in the USA [45]. Possible contributing factors that have earlier been linked to SES include screening participation [64], treatment approach [65–68], and surgical procedure [69]. For example, a low rate of minimally invasive surgery compared to open surgery was observed in patients with lower SES, as well as a higher conversion rate [69]. No differences in the distribution of laparoscopic, robotic, or open procedures, or in conversion rates, were observed in our study cohort. Furthermore, no statistically significant association between area-level SES and treatment approach in terms of neoadjuvant treatment was observed in our patient cohort, in contrast to previous literature [66]. Lifestyle factors considered prognostic for the occurrence of CRC, such as smoking and increased comorbidities, which are typically associated with lower SES, were not associated with lower area-level SES in our study cohort. Earlier data originate from countries both with and without universal healthcare systems, which are designed to provide patients with equal access to diagnostics and treatment. This suggests that a universal healthcare system alone is not the decisive factor influencing survival in CRC patients.

Data on the relationship between CRC outcomes and SES in Germany are still limited. Available German studies have largely been based on population-based registry data [43, 44], whereas our study provides a more detailed clinical perspective from a contemporary surgical cohort with detailed perioperative and postoperative information. Jansen et al. show SES inequalities in CRC survival based on three population-based clinical cancer registries in Germany [43]. In contrast, lower area-level SES was not significantly associated with worse overall or disease-free survival in our cohort, thereby adding an important perspective to the existing literature. Our study was conducted within the German healthcare system, in which universal health insurance coverage has been mandatory since 2009, and at a high-volume certified colorectal cancer center, where standardized diagnostic and treatment pathways may reduce disparities in access to cancer care and thereby attenuate SES-related differences in oncologic outcomes.

The present study has several limitations that should be considered when interpreting the findings. First, as a single-center study, it is restricted to one region, where differences may be less pronounced due to relatively homogeneous healthcare structures. However, the majority of our patient population was drawn from the Hamburg Metropolitan Region, which extends across Hamburg, Lower Saxony, Schleswig–Holstein, and Mecklenburg-Western Pomerania and comprises approximately 5.5 million inhabitants. This broader catchment area covers a wide range of socioeconomic strata and may therefore partially mitigate the limitation of a geographically restricted study population. Second, the study was conducted at a high-volume certified colorectal cancer center in Germany, which may limit the generalizability of the findings to other hospital settings and healthcare systems outside the German context. In addition, the postal-code based PPI used in this study reflects area-level socioeconomic conditions. Other individual dimensions of SES, such as education, occupation, insurance status, and screening behavior, were not taken into account in the present analysis. Finally, owing to the retrospective single-center design, the possibility of selection bias and residual confounding remains.

In conclusion, no statistically significant association was observed between low area-level SES and poorer oncologic outcomes after colorectal cancer surgery in this cohort. In the overall cohort, higher area-level SES was associated with more frequent emergency presentations. The top SES subgroup was further associated with older age and a higher rate of major postoperative complications. Patients in the bottom SES subgroup were significantly younger and had a higher BMI at diagnosis.

## Supplementary Information

Below is the link to the electronic supplementary material.

Supplementary file1 (DOCX 275 KB)

## Data Availability

The data that support the findings of this study are available from the corresponding author upon reasonable request.
