# Supplementary material for: Impact of socioeconomic status on patient characteristics and postoperative outcomes in colorectal cancer surgery: A retrospective cohort study
Source: Int J Colorectal Dis. 2026 May 25;41(1):91. doi: 10.1007/s00384-026-05149-z (PMC13201314; doi:10.1007/s00384-026-05149-z)
Supplement: Supplementary file 1 — Supplementary file1 (DOCX 275 KB) [file 384_2026_5149_MOESM1_ESM.docx]

**
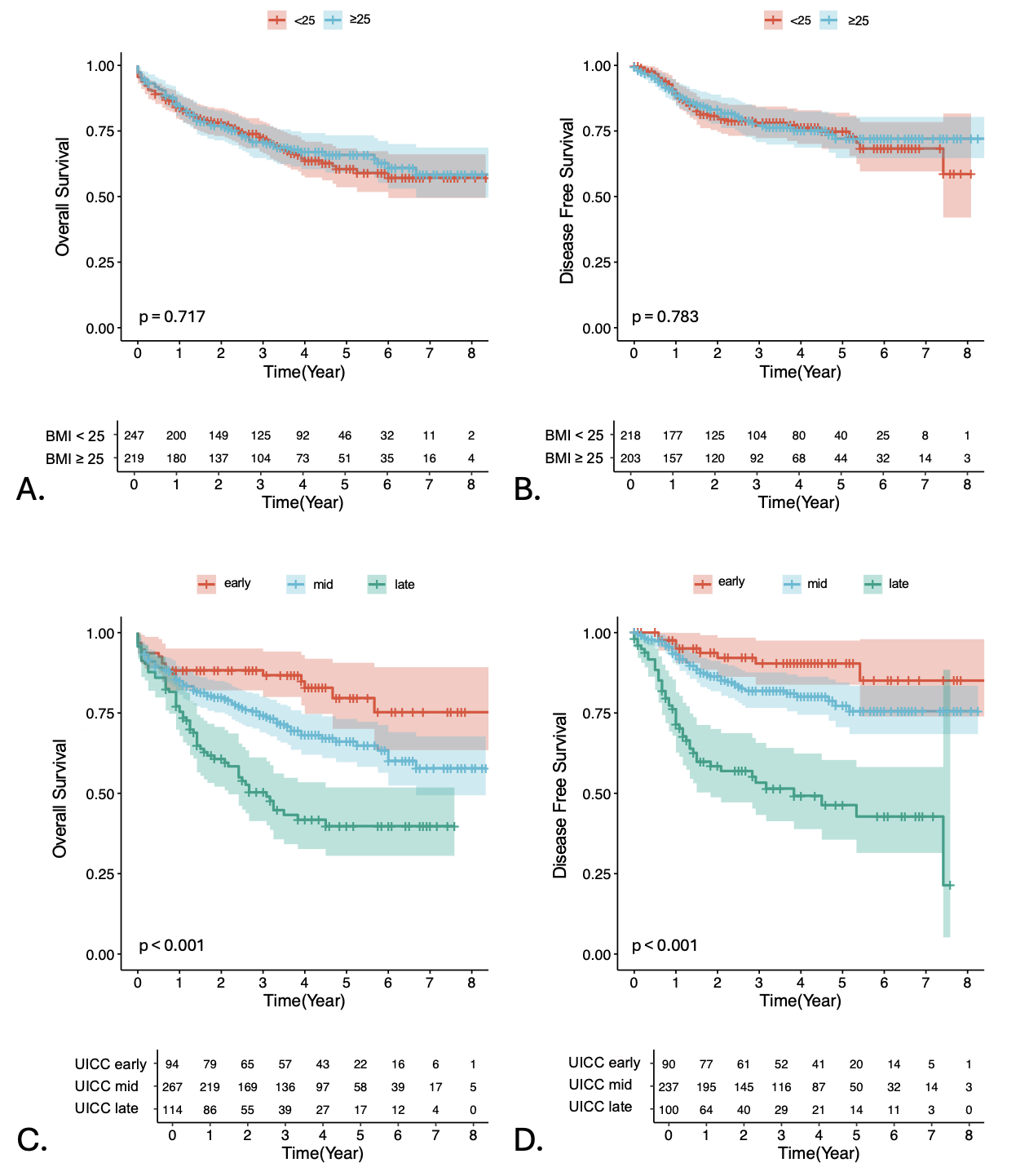
**

**Supplementary Figure 1.** The Kaplan-Meier survival curves show the association between BMI and overall survival (A.) and disease-free survival (B.), as well as between UICC stage and overall survival (C.) and disease-free survival (D.) in patients with colorectal cancer.

**Supplementary Table 1. Overview of Variables collected in the REDCap Database**

| **Category** | **Variables** |
| --- | --- |
| Demographics | Record ID; gender; date of birth; weight (kg); height (cm); postal code; purchasing power |
| Comorbidities and medical history | ASA physical status; ECOG performance status; heart failure; NYHA class; coronary artery disease; previous myocardial infarction; peripheral vascular disease; cerebrovascular event / TIA; dementia; current or former smoking; pack-years; COPD; GOLD stage; liver disease; Child–Pugh score; inflammatory bowel disease; alcohol abuse; diabetes mellitus; hemiplegia; moderate to severe chronic kidney disease; connective tissue disease; peptic ulcer disease; solid tumor; leukemia; lymphoma; AIDS; immunosuppressive therapy or long-term corticosteroid use; immunosuppressive agent / corticosteroid regimen; anticoagulant therapy; anticoagulant agent; previous abdominal surgery; previous abdominal surgery – specification; previous malignancy; previous malignancy – entity/status |
| Laboratory parameters | Hb; leukocytes; CRP; bilirubin; creatinine; albumin; INR; CEA; CA 19-9 |
| Preoperative diagnostics | Digital rectal examination; rigid rectoscopy; colonoscopy; histological confirmation; endoscopic tattooing; abdominal CT; chest CT; pelvic MRI; contrast-enhanced liver ultrasound; Primovist-enhanced liver MRI |
| Operative details | Urgency; primary surgeon; surgical approach; conversion; reason for conversion – specification; procedure; protective ileostomy; time of incision; time of skin closure; anastomotic technique; liver resection – specification; resection of other organ – specification; intraoperative blood loss (mL); number of packed red blood cell units; number of fresh frozen plasma units |
| Tumor characteristics and pathology | Tumor location; Sample number; histological entity; UICC stage; grading; pT stage; pN stage; pM stage; number of resected lymph nodes; number of positive lymph nodes; resection status; location of R1/R2 resection – specification; MERCURY classification (rectal cancer); WEST classification (colon cancer); microsatellite status; BRAF V600 mutation status; RAS mutation status |
| Neoadjuvant and adjuvant therapy | Neoadjuvant chemotherapy; neoadjuvant chemotherapy – protocol; neoadjuvant chemotherapy – number of cycles; neoadjuvant radiotherapy; neoadjuvant radiotherapy dose; adjuvant chemotherapy; adjuvant chemotherapy – protocol; adjuvant chemotherapy – number of cycles; adjuvant radiotherapy; adjuvant radiotherapy dose |
| Perioperative outcomes and postoperative complications | Acute aspiration; pneumonia; pleural effusion requiring drainage; respiratory failure requiring reintubation; acute respiratory distress syndrome; cardiac arrest requiring cardiopulmonary resuscitation; ST-segment elevation myocardial infarction; non-ST-segment elevation myocardial infarction; atrial arrhythmia; ventricular arrhythmia; anastomotic leakage; anastomotic bleeding; mechanical ileus requiring reoperation; Clostridioides difficile infection; mesenteric ischemia requiring reoperation; intestinal perforation requiring reoperation; acute renal failure; urinary tract infection; deep venous thrombosis; pulmonary embolism; stroke; positioning-related nerve injury; acute delirium; wound infection; generalized sepsis; abdominal wall dehiscence; chyle leak; reoperation for postoperative bleeding; Clavien–Dindo classification; discharge status; date of discharge |
| Oncological outcomes and follow-up | Cancer recurrence – date; cancer recurrence – specification; death status; date of death; date of last follow-up |

Abbreviations: AIDS, acquired immunodeficiency syndrome; ASA, American Society of Anesthesiologists; BRAF, v-Raf murine sarcoma viral oncogene homolog B; CA 19-9, carbohydrate antigen 19-9; CEA, carcinoembryonic antigen; COPD, chronic obstructive pulmonary disease; CRP, C-reactive protein; CT, computed tomography; ECOG, Eastern Cooperative Oncology Group; GOLD, Global Initiative for Chronic Obstructive Lung Disease; Hb, hemoglobin; INR, international normalized ratio; MRI, magnetic resonance imaging; NYHA, New York Heart Association; RAS, rat sarcoma viral oncogene homolog; TIA, transient ischemic attack; UICC, Union for International Cancer Control.

**Supplementary Table 2. Summary of missing data in the overall cohort (Table 1)**

| **Variables** | **Missing (n)** | **Available (n)** |
| --- | --- | --- |
| Record ID | 0 | 476 |
| Age | 0 | 476 |
| Gender | 0 | 476 |
| Purchasing Power | 0 | 476 |
| BMI | 9 | 467 |
| Nicotine abuse | 0 | 476 |
| Neoadjuvant Chemotherapy | 0 | 476 |
| Neoadjuvant Radiotherapy | 0 | 476 |
| Charlson Comorbidity Index | 0 | 476 |
| Surgical approach | 0 | 476 |
| UICC | 0 | 476 |
| pT | 0 | 476 |
| pN | 0 | 476 |
| pM | 1 | 475 |
| Resection Status | 12 | 464 |
| Clavien-Dindo Classification | 2 | 474 |
| Adjuvant Chemotherapy | 0 | 476 |
| Adjuvant Radiotherapy | 0 | 476 |
| Tumor Location | 0 | 476 |
| ECOG | 18 | 458 |
| Diabetes | 0 | 476 |
| Urgency of surgery | 0 | 476 |
| Conversion | 0 | 476 |
| Procedure | 1 | 475 |
| Operative Time | 1 | 475 |
| ASA | 9 | 467 |
| Length of hospital stay | 35 | 441 |

Abbreviations: ASA, American Society of Anesthesiologists; BMI, body mass index; ECOG, Eastern Cooperative Oncology Group; UICC, Union for International Cancer Control.

**Supplementary Table 3. Summary of missing data in the top and bottom SES subgroups (Table 3)**

| **Variable** | **Missing (n)** | **Available (n)** |
| --- | --- | --- |
| Record ID | 0 | 190 |
| Age | 0 | 190 |
| Gender | 0 | 190 |
| Purchasing Power | 0 | 190 |
| BMI | 3 | 187 |
| Nicotine abuse | 0 | 190 |
| Neoadjuvant Chemotherapy | 0 | 190 |
| Neoadjuvant Radiotherapy | 0 | 190 |
| Charlson Comorbidity Index | 0 | 190 |
| Surgical approach | 0 | 190 |
| UICC | 0 | 190 |
| pT | 0 | 190 |
| pN | 0 | 190 |
| pM | 0 | 190 |
| Resection Status | 7 | 183 |
| Clavien-Dindo Classification | 1 | 189 |
| Adjuvant Chemotherapy | 0 | 190 |
| Adjuvant Radiotherapy | 0 | 190 |
| Tumor Location | 0 | 190 |
| ECOG | 10 | 180 |
| Diabetes | 0 | 190 |
| Urgency of surgery | 0 | 190 |
| Conversion | 0 | 190 |
| Procedure | 1 | 189 |
| Operative Time | 0 | 190 |
| ASA | 1 | 189 |
| Length of hospital stay | 15 | 175 |

Abbreviations: ASA, American Society of Anesthesiologists; BMI, body mass index; ECOG, Eastern Cooperative Oncology Group; UICC, Union for International Cancer Control.

**Supplementary Table 4. Multivariable logistic regression analysis of emergency surgery in the overall cohort, with socioeconomic status, age and Charlson Comorbidity Index as independent variables**

| **Characteristics** | **Odds Ratio** | **95% CI** | ***p*-value** |
| --- | --- | --- | --- |
| Age | 1 | 0.98 - 1.03 | 0.74 |
| Charlson Comorbidity Index | 1,09 | 0.95 - 1.24 | 0.18 |
| High SES | 2,15 | 1.24 - 3.87 | 0.01 |

**Supplementary Table 5. Multivariable logistic regression analysis of postoperative complications in the overall cohort, with socioeconomic status, age and Charlson Comorbidity Index as independent variables**

| **Characteristics** | **Odds Ratio** | **95% CI** | ***p*-value** |
| --- | --- | --- | --- |
| Age | 1,02 | 0.99 - 1.04 | 0,24 |
| Charlson Comorbidity Index | 1,04 | 0.9 - 1.21 | 0,6 |
| High SES | 1,31 | 0.78 - 2.19 | 0,31 |

**Supplementary Table 6. Multivariable logistic regression analysis of emergency surgery in the top and bottom SES subgroups, with socioeconomic status, age, and Charlson Comorbidity Index as independent variables**

| **Characteristics** | **Odds Ratio** | **95% CI** | ***p*-value** |
| --- | --- | --- | --- |
| Age | 1 | 0.96 - 1.03 | 0.79 |
| Charlson Comorbidity Index | 1,03 | 0.82 - 1.25 | 0.8 |
| Top SES | 5,05 | 2.15 - 13.38 | <0.001 |

**Supplementary Table 7. Multivariable logistic regression analysis of postoperative complications in the top and bottom SES subgroups, with socioeconomic status, age, and Charlson Comorbidity Index as independent variables**

| **Characteristics** | **Odds Ratio** | **95% CI** | ***p*-value** |
| --- | --- | --- | --- |
| Age | 0,99 | 0.95 - 1.02 | 0,5 |
| Charlson Comorbidity Index | 1,1 | 0.9 - 1.38 | 0,39 |
| Top SES | 1,29 | 0.56 - 3.01 | 0,55 |
